# Northern Infirmary, Inverness

**Published:** 1905-11-25

**Authors:** 


### NORTHERN INFIRMARY, INVERNESS.

### THE UNIFORM SYSTEM ADOPTED.

We congratulate the Treasurer (Mr. Charles M. Brown) and the Committee of the Northern Infirmary, Inverness, upon the fact that during 1904 the books of this institution have been placed on the uniform system. We have received a copy of the first report in which these accounts appear, and it is satisfactory to observe the clear and ample way in which the whole of the accounts are now rendered. The number of patients treated in the Infirmary in 1904 was 854, an increase of 185 on the previous year. The average daily number of in-patients was 87, as against 76 in 1903. The average duration of treatment of each in-patient was 30.3 days, as against 33.4 days in 1903. It would appear from the latter figures that a number of relatively chronic cases are admitted to treatment, or that patients after operation are retained through the convalescent stage and are not sent out until they are fit to resume their ordinary occupations. Otherwise the average days' residence for in-patients would be unusually high. It is an interesting feature of this Infirmary that 761 out of 854 in-patients were admitted on the application of members of the medical profession. This fact would seem to prove that the relations between the general practitioners and the Northern Infirmary staff are very cordial and satisfactory.

